# Molecular Typing of *Campylobacter jejuni* and *Campylobacter coli* Isolated from Various Retail Meats by MLST and PFGE

**DOI:** 10.3390/foods3010082

**Published:** 2014-01-08

**Authors:** Aneesa Noormohamed, Mohamed K. Fakhr

**Affiliations:** Department of Biological Science, The University of Tulsa, Tulsa, OK 74104, USA; E-Mail: aneesa-noormohamed@utulsa.edu

**Keywords:** *Campylobacter*, MLST, PFGE, molecular typing, retail meats, poultry, beef, pork, livers, foodborne pathogens

## Abstract

*Campylobacter* species are one of the leading causes of foodborne disease in the United States. *Campylobacter jejuni* and *Campylobacter coli* are the two main species of concern to human health and cause approximately 95% of human infections. Molecular typing methods, such as pulsed-field gel electrophoresis (PFGE) and multilocus sequence typing (MLST) are often used to source track foodborne bacterial pathogens. The aim of the present study was to compare PFGE and MLST in typing strains of *C. jejuni* and *C. coli* that were isolated from different Oklahoma retail meat sources. A total of 47 *Campylobacter* isolates (28 *C. jejuni* and 19 *C. coli*) isolated from various retail meat samples (beef, beef livers, pork, chicken, turkey, chicken livers, and chicken gizzards) were subjected to pulsed-field gel electrophoresis (PFGE) and multilocus sequence typing (MLST). PFGE was able to group the 47 *Campylobacter* isolates into two major clusters (one for *C. jejuni* and one for *C. coli*) but failed to differentiate the isolates according to their source. MLST revealed 21 different sequence types (STs) that belonged to eight different clonal complexes. Twelve of the screened *Campylobacter* isolates (8 *C. jejuni* and 4 *C. coli*) did not show any defined STs. All the defined STs of *C. coli* isolates belonged to ST-828 complex. The majority of *C. jejuni* isolates belonged to ST-353, ST-607, ST-52, ST-61, and ST-21 complexes. It is worthy to mention that, while the majority of *Campylobacter* isolates in this study showed STs that are commonly associated with human infections along with other sources, most of the STs from chicken livers were solely reported in human cases. In conclusion, retail meat *Campylobacter* isolates tested in this study particularly those from chicken livers showed relatedness to STs commonly associated with humans. Molecular typing, particularly MLST, proved to be a helpful tool in suggesting this relatedness to *Campylobacter* human isolates.

## 1. Introduction

*Campylobacter* is a foodborne pathogen that is one of the leading causes of bacterial gastroenteritis [1]. It causes an estimated 1.3 million infections a year [1]. It is the third most common cause of bacterial foodborne illness in the United States, after *Salmonella* [1]. The most common species isolated are *Campylobacter jejuni* and *Campylobacter coli*, which, together, cause around 95% of all *Campylobacter* infections [2,3]. Contaminated food is the most common mode of infection with *Campylobacter*. The most common food source is poultry [4].

Molecular typing is used to differentiate between isolates of the same species of bacteria [5]. Genotyping methods can be used to identify the genetic relatedness between different strains of bacteria. In order to track *Campylobacter* infections, various genotyping methods are used, such as pulsed-field gel electrophoresis (PFGE) and multilocus sequence typing (MLST). PFGE is based on gel electrophoresis of restriction digested genomic DNA. Traditional gel electrophoresis has a constant current in one direction so only small fragments can enter the gel and be separated. In PFGE, the direction of current changes regularly (pulsed) and, thus, large fragments twist and move slowly through the gel [6,7]. The pattern of the bands determines the relatedness of the isolates. PFGE is considered the “gold standard” in molecular typing for most bacteria, including foodborne pathogens, as the entire genome of the microbe is analyzed to create restriction profiles [8,9], however, it has its disadvantages in that it requires expensive equipment and complicated protocols, in addition to which, there are no standard methods for the interpretation of data, or sharing of this data with other scientists [9,10,11]. In fact, the genetic variation among *Campylobacter* becomes a concern when using PFGE for genotyping [12]. Some strains are not typable using either of the commonly used restriction enzymes *Sma*I or *Kpn*I, which bring about questions as to the usefulness of PFGE with *Campylobacter* species [11,13].

MLST typing is based on gene sequences of seven selected genes, which are considered “housekeeping” genes. These genes are selected as they are fairly conserved. For each gene, each recorded sequence is given a number. The resulting seven-digit number defines the isolate. The isolates with related sequence types (STs) can also be grouped together into clonal complexes. There may be minor differences between “identical” MLST isolates (e.g., from the PFGE pattern). In the case of MLST, there is a useful website that has, not only *Campylobacter* MLST primers, but it also allows scientists to input the gene sequences, which can then be accessed by scientists worldwide to compare, and the protocols are not as involved [14]. MLST also has the discriminatory power to characterize hypervariable genomes, such as those of *Campylobacte*r [15], although it isn′t able to separate closely-related isolates [16,17]. More recently, developed MLST protocols to study several different bacterial pathogens became available [18,19]. The MLST schemes for *C. jejuni* and *C. coli* have previously been determined [14,20] and are available on the MLST website [21]. The MLST website also carries information on several different sequence types and is used to share this information with scientists worldwide. Alternate schemes that do not use all the same genes are also available [22,23].

The objective of this study was to determine the genetic relatedness among 47 strains of *C. jejuni* and *C. coli*, isolated from different retail meat sources and to determine if one typing method is superior to the other one in determining such relatedness.

## 2. Experimental Section

### 2.1. Bacterial Isolates

Forty-seven *C. jejuni* and *C. coli*, previously isolated from retail meat samples, were used in this study for MLST and PFGE [24,25]. The isolates were selected to represent both species (*C. jejuni* and *C. coli*), several meat brands, as well as different retail meat sources, such as chicken (breast and thighs), turkey (breast, thighs, neck pieces, and ground), beef livers, pork (tongue), chicken livers, and chicken gizzards (Table 1). All isolates were kept frozen at −80 °C in Brucella broth (Becton Dickinson, Sparks, MD, USA) with 20% glycerol.

**Table 1:** Number and sources of isolates for each *Campylobacter* species used in this study.

Source	*C. jejuni*	*C. coli*
Chicken	7	3
Chicken Livers	4	5
Chicken Gizzards	7	2
Turkey	5	2
Beef Livers	5	5
Pork	0	2
Total	28	19

### 2.2. Pulsed-Field Gel Electrophoresis Typing

The isolates were typed by PFGE following the PulseNet protocol for *Campylobacter* [26]. Briefly, isolates were grown on MH agar with 5% laked-horse blood and then diluted to the required concentration and agarose-embedded plugs were made and washed. They were then digested with *SmaI* restriction enzyme (Promega, Madison, WI, USA). The digested plugs were run in Seakem agarose gel (Lonza, Allendale, NJ, USA) with 0.5× Tris-Borate EDTA (TBE) buffer (Amresco, Solon, OH, USA) to separate the bands on the CHEF Mapper PFGE system (Bio-Rad) by running for 16 h at 14 °C switching directions every 6.76 s and ending with 35.38 s (25). *Salmonella enterica* serovar Braenderup digested with *XbaI* (Promega, Madison, WI, USA) was used as the molecular reference marker. Gels were stained with ethidium bromide and viewed and recorded under UV transillumination (UVP, Upland, CA, USA). Gel images were analyzed using BioNumerics software (Applied Maths, Austin, TX, USA). The banding patterns were clustered using Dice coefficients using unweighted pair group method, with arithmetic mean (UPGMA), and a 3% band tolerance.

### 2.3. Multilocus Sequence Typing

MLST was performed for the same 47 isolates that were typed in the PFGE study. PCR for each of the following seven housekeeping genes was performed: *aspA* (aspartase A), *glnA* (glutamine synthetase), *gltA* (citrate synthase), *glyA* (serine hydroxymethyltransferase), *pgm* (phosphoglucomutase), *tkt* (transketolase), and *uncA* (ATP synthase α subunit) [14,27]. Bacterial DNA extracts used in polymerase chain reaction (PCR) were prepared from *Campylobacter* cultures using the single cell lysing buffer (SCLB) method [28].

The selected isolates were tested for the presence of the seven different housekeeping genes used in the MLST scheme for *C. jejuni* by PCR reactions. The primers used were available at the MLST website [29,30] and are shown in Table 2. The PCR was carried out in 25 µL reactions. Each 25 µL reaction contained 12.5 µL GoTaq^®^ Green Master Mix (Promega, Madison, WI, USA), 3.5 µL sterile water (Promega, Madison, WI, USA), 1 µL (25 pmol) each primer (IDT, Coralville, IA, USA), and 3 µL of template DNA. The cycling conditions were set as follows: (1) 95 °C for 5 min; (2) 94 °C for 1 min; (3) 50 °C for 1 min; (4) 72 °C for 1 min; and (5) 72 °C for 10 min. Steps 2 through 4 were repeated for 35 cycles. Once the cycles were complete, reactions were held at 4 °C until gel electrophoresis. Ten microliters of PCR product was subjected to horizontal electrophoresis in a 1% agarose gel in 1× Tris-acetate-EDTA (TAE) buffer. A 1 kb plus ladder (Bioneer, Alameda, CA, USA) was used as the molecular marker. Gels were viewed and recorded by ultraviolet transillumination, using a UV imager (UVP). Sterile water was used as the negative control.

The PCR products were purified using ExoSAP-IT enzyme (Affymetrix, Santa Clara, CA, USA). The sequencing PCR reaction was prepared according to a modified ABI 3130*xl* manufacturer′s sequencing protocol (Applied Biosystems, Foster City, CA, USA). Briefly, sequencing reactions were prepared to a 15 µL volume containing 3.5 µL purified PCR product, 1.5 μL primer, 0.5 μL sequencing buffer, 2 μL betaine, 0.5 μL BigDye, and 2 μL RNase-free water. The cycling conditions were set up according to the ABI capillary sequencer instructions (Applied Biosystems, Foster City, CA, USA). The sequenced products were then read using the ABI 3130*xl* (Applied Biosystems) and analyzed using BioNumerics software (Applied Maths, Austin, TX, USA), which has a function to determine STs and clonal complexes by directly submitting the sequences to the MLST website.

**Table 2:** Polymerase chain reaction (PCR) and sequencing primer sets for the multilocus sequence typing (MLST) scheme for *C. jejuni* and *C. coli*.

Genes	Primer Sequences	Use	References
*aspA*	5′-AGTACTAATGATGCTTATCC-3′ 5′-ATTTCATCAATTTGTTCTTTGC-3′	*C. jejuni* PCR	[14]
*glnA*	5′-TAGGAACTTGGCATCATATTACC-3′ 5′-TTGGACGAGCTTCTACTGGC-3′	*C. jejuni* PCR	[14]
*gltA*	5′-GGGCTTGACTTCTACAGCTACTTG-3′ 5′-CCAAATAAAGTTGTCTTGGACGG-3′	*C. jejuni* PCR	[14]
*glyA*	5′-GAGTTAGAGCGTCAATGTGAAGG-3′ 5′-AAACCTCTGGCAGTAAGGGC-3′	*C. jejuni* PCR	[14]
*pgm*	5′-TACTAATAATATCTTAGTAGG-3′ 5′-CACAACATTTTTCATTTCTTTTTC-3′	*C. jejuni* PCR	[14]
*tkt*	5′-GCAAACTCAGGACACCCAGG-3′ 5′-AAAGCATTGTTAATGGCTGC-3′	*C. jejuni* PCR	[14]
*uncA*	5′-ATGGACTTAAGAATATTATGGC-3′ 5′-GCTAAGCGGAGAATAAGGTGG-3′	*C. jejuni* PCR	[14]
*aspA*	5′-AGTACTAATGATGCTTATCC-3′ 5′-ATTTCATCAATTTGTTCTTTGC-3′	*C. jejuni* sequencing	[14]
*glnA*	5′-TAGGAACTTGGCATCATATTACC-3′ 5′-TTGGACGAGCTTCTACTGGC-3′	*C. jejuni* sequencing	[14]
*gltA*	5′-GGGCTTGACTTCTACAGCTACTTG-3′ 5′-CCAAATAAAGTTGTCTTGGACGG-3′	*C. jejuni* sequencing	[14]
*glyA*	5′-GAGTTAGAGCGTCAATGTGAAGG-3′ 5′-AAACCTCTGGCAGTAAGGGC-3′	*C. jejuni* sequencing	[14]
*pgm*	5′-TACTAATAATATCTTAGTAGG-3′ 5′-CACAACATTTTTCATTTCTTTTTC-3′	*C. jejuni* sequencing	[14]
*tkt*	5′-GCAAACTCAGGACACCCAGG-3′ 5′-AAAGCATTGTTAATGGCTGC-3′	*C. jejuni* sequencing	[14]
*uncA*	5′-ATGGACTTAAGAATATTATGGC-3′ 5′-GCTAAGCGGAGAATAAGGTGG-3′	*C. jejuni* sequencing	[14]
*aspA*	5′-CCAACTGCAAGATGCTGTACC-3′ 5′-TTCATTTGCGGTAATACCATC-3′	*C. coli* PCR and sequencing	[27]
*glnA*	5′-CATGCAATCAATGAAGAAAC-3′ 5′-TTCCATAAGCTCATATGAAC-3′	*C. coli* PCR and sequencing	[27]
*gltA*	5′-CTTATATTGATGGAGAAAATGG-3′ 5′-CCAAAGCGCACCAATACCTG-3′	*C. coli* PCR and sequencing	[27]
*glyA*	5′-AGCTAATCAAGGTGTTTATGCGG-3′ 5′-AGGTGATTATCCGTTCCATCGC-3′	*C. coli* PCR and sequencing	[27]
*pgm*	5′-GGTTTTAGATGTGGCTCATG-3′ 5′-TCCAGAATAGCGAAATAAGG-3′	*C. coli* PCR and sequencing	[27]
*tkt*	5′-GCTTAGCAGATATTTTAAGTG-3′ 5′-AAGCCTGCTTGTTCTTTGGC-3′	*C. coli* PCR and sequencing	[27]
*uncA*	5′-AAAGTACAGTGGCACAAGTGG-3′ 5′-TGCCTCATCTAAATCACTAGC-3′	*C. coli* PCR and sequencing	[27]

## 3. Results and Discussion

### 3.1. Pulsed-Field Gel Electrophoresis

**Figure 1:**
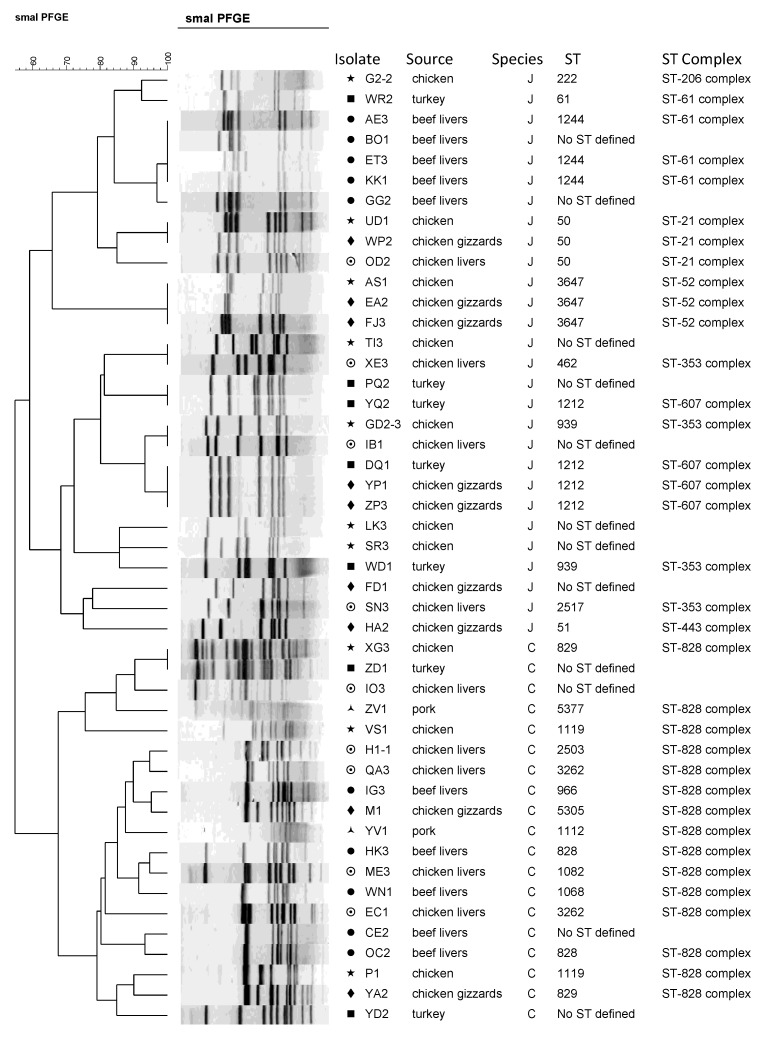
The MLST and PFGE profile comparison of the *Campylobacter jejuni* and *Campylobacter coli* isolates. Symbols represent the different sources of the isolates. No ST defined means that no sequence type was identified for that particular isolate. ST, sequence type; ST complex, MLST clonal complex.

The results of the PFGE showed that the isolates studied were separated into two major groups according to their species (*C. jejuni* and *C. coli*) (Figure 1). The isolates were also able to be separated by their ST-complexes within the species groups. PFGE was able to group the 47 isolates into 2 major clusters (one for *C. jejuni* and one for *C. coli*) but wasn’t able to differentiate the isolates by meat source within the species (Figure 1). By PFGE separation, the isolates were found to also cluster into their ST-complexes, such as for ST-61, ST-21, ST-52 for *C. jejuni*, and ST-828 for *C. coli* (Figure 1). Among the *C. jejuni* isolates, ST-61 was related to beef liver isolates and ST-21 and ST-52 related to chicken sources. ST-607 isolates did not all cluster closely together but they all belonged to poultry sources and to the same ST (1212). ST-353 isolates also did not cluster together, but they all belonged to poultry sources (Figure 1). When using PFGE for genotyping, genetic variation among *Campylobacter* strains becomes a concern [12]. Some strains are not typable, using either of the commonly used restriction enzymes *Sma*I or *Kpn*I, which creates questions about the usefulness of PFGE with *Campylobacter* species [11,13]. In our study PFGE was able to group the 47 *Campylobacter* isolates into two major clusters (one for *C. jejuni* and one for *C. coli*) but failed to differentiate the isolates according to their source.

### 3.2. Multilocus Sequence Typing

**Figure 2:**
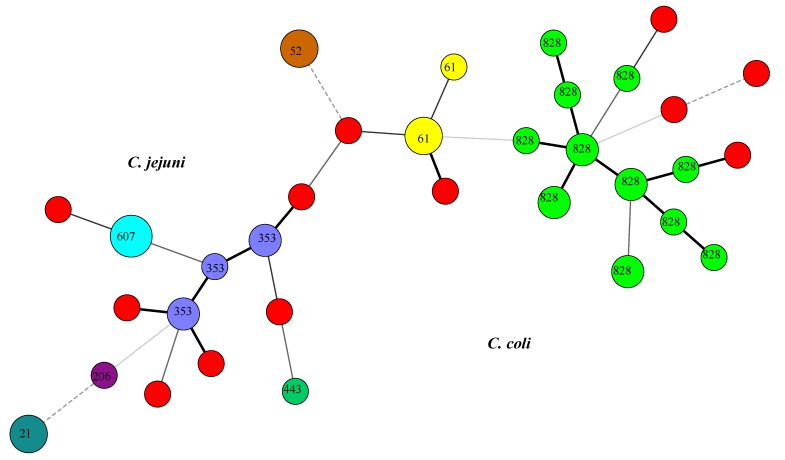
Minimum spanning tree showing the clustering of the *Campylobacter* STs showing the two species type clusters. Each circle represents a clonal complex. The number inside each circle is the clonal complex. Red circles denote isolates that were not defined. The size of the circle is proportional to the number of strains represented. Thick lines denote closer association between the groups and thin lines denote less. The dashed lines denote least association between members.

MLST was able to separate the isolates into 21 different STs, which belonged to eight different clonal complexes (Figure 1). Twelve of the isolates (eight *C. jejuni* and four *C. coli*) were not assigned STs (Figure 1). All the defined STs of *C. coli* isolates belonged to ST-828 complex. The majority of the *C. jejuni* isolates grouped into ST-353, ST-607, ST-52, and ST-61 clonal complexes (Figure 1). The most common clonal complex observed in this study was the ST-828 complex, which consists mostly of *C. coli* isolates. Other clonal complexes identified were ST-21, ST-61, ST-52, ST-206, ST-353, ST-443, and ST-607. Cluster analysis by Minimum Spanning Tree created using the BioNumerics software also shows that the isolates clustered into two distinct groups according to their species, which are *C. jejuni* and *C. coli* (Figure 2). *Campylobacter jejuni* appears to be more diverse than *Campylobacter coli* in regards to their STs and clonal complexes distributions (Figure 2).

Figure 2 shows the separation of the isolates by MLST according to their ST-complexes.The isolates were separated according to the species of *Campylobacter* they belong to and, within those groups, there is a separation into ST-complexes. The unidentified isolates were also added and show affiliation with one group or the other. The most common clonal complex was ST-828 consisting of *C. coli* isolates, which has been previously observed [11,17,27,31,32]. ST-828 was also reported as the most common complex among *C. coli* isolates by other studies [11,27,31,32,33,34]. This was also the case with the *C. coli* isolates in this study for all of our defined isolates. Most human infections are caused by complexes ST-828 and ST-1150 [17]. In fact, the ST-828 complex is commonly associated with human isolates, as well as chicken meat or offal, according to data from the MLST website [17]. ST-21 has also been reported to be the most commonly detected complex among the isolates that were published on the MLST website with the most isolates from human origins [17], and the next most common belong to chicken isolates [17,35,36]. ST-21 has also been found in bovine and ovine isolates [37,38]. Chicken is also the source of ST-52, ST-61, ST-206, ST-353, and ST-828 in other studies [5]. In our study, all of these ST complexes belonged to various poultry isolates. ST-353 and ST-21 were also previously reported among *C. jejuni* isolates [5,34,37,38]. Colles *et al.*, 2003 [39], reported in their study that the ST-61 complex was associated with sheep isolates. Data collected from the MLST website in 2012 by Colles and Maiden [17], found that ST-61 was most commonly associated with human isolates and the next most common source was beef offal or meat. ST-206 was also most associated with human isolates in that study [17].

The fact that all the *C. coli* isolates belong to the same complex could be due to the more conservative nature of the *C. coli* genome and the *C. jejuni* genome being more variable [27,40]. Colles *et al.* [39], and Manning *et al.* [22], found that there was no association of the STs with the host, inferring that this could be due to the lack of diversity among *C. coli* or possibly due to their sample not being very diverse. Miller *et al.* [31], reported that, in their MLST studies, there was some association among *C. coli* and their STs to specific hosts suggesting that source tracking would be possible with *C. coli*.

Most of the STs identified in this study were found to be associated with human and other sources of infection. Most of the STs found in the chicken livers were STs associated with human infection only according to the MLST website [29]. Adding to the importance of chicken livers as a public health risk is the recent discovery by Strachan *et al*. [41], that molecular source attribution by MLST demonstrated that *Campylobacter* strains from chicken livers were most similar to those found commonly in humans, which provides further evidence that chicken liver is a probable source of human infection.

## 4. Conclusions

In conclusion, retail meat *Campylobacter* isolates tested in this study particularly those from chicken livers showed relatedness to STs commonly associated with humans. Molecular typing particularly MLST proved to be a helpful tool in suggesting this relatedness to *Campylobacter* human isolates and can be regarded as superior to PFGE in this regard. The genetic variation among *C. jejuni* strains appeared higher than that among *C. coli* strains using MLST in our study.
